# Time-of-arrival detection for time-resolved scanning transmission X-ray microscopy imaging

**DOI:** 10.1107/S1600577520007262

**Published:** 2020-07-14

**Authors:** Simone Finizio, Sina Mayr, Jörg Raabe

**Affiliations:** a Paul Scherrer Institut, Forschungsstrasse 111, 5232 Villigen PSI, Switzerland; b Laboratory for Mesoscopic Systems, Department of Materials, ETH Zurich, 8093 Zurich, Switzerland

**Keywords:** STXM, time-resolved imaging, synchrotron light source

## Abstract

A setup for time-resolved scanning transmission X-ray microscopy imaging is presented, which allows for an increase in the temporal resolution without the requirement of operating the synchrotron light source with low-α optics through the measurement of the time-of-arrival of the X-ray photons. Measurements of two filling patterns in hybrid mode of the Swiss Light Source are presented as a first proof-of-principle and benchmark for the performances of this new setup. From these measurements, a temporal resolution on the order of 20–30 ps could be determined.

## Abstract 2

A new method for time-resolved scanning transmission X-ray microscopy imaging, to measure the time-of-arrival of the X-ray photons, is presented. This method promises an improvement in the time resolutions achievable with normal optics filling patterns.

Time-resolved imaging, using the pump–probe protocol, is a powerful technique for the investigation of dynamical processes occurring in many materials studied in condensed matter research. Combined with a suitable microscopy technique such as photoemission electron microscopy or scanning transmission X-ray microscopy, sub-100 nm spatial and sub-nanosecond temporal resolutions are routinely accessible. A particularly successful application of this technique is the investigation of time-resolved magneto-dynamical processes occurring at the sub-nanosecond timescale using time-resolved scanning transmission X-ray microscopy (TR-STXM) at the soft X-ray energy range. Examples include investigation of the nucleation, annihilation and manipulation of topological objects such as magnetic vortices and skyrmions (Finizio *et al.*, 2017 ▸, 2019*b* ▸; Kammerer *et al.*, 2011 ▸; Litzius *et al.*, 2017 ▸; Woo *et al.*, 2018 ▸), the manipulation of magnetic domains and domain walls (Bisig *et al.*, 2013 ▸; Finizio *et al.*, 2019*a* ▸), and the generation and propagation of spin waves (Wintz *et al.*, 2016 ▸; Förster *et al.*, 2019 ▸; Dieterle *et al.*, 2019 ▸). For the majority of these studies, a frequency range of up to 10 GHz is usually required (and accessible). However, new investigations, *e.g.* on antiferromagnetic spintronics (Marrows, 2006 ▸), will push this limit towards frequencies of several tens of GHz, prompting the development of measurement techniques able to tackle this new requirement.

STXM is a transmission microscopy technique where the spatially resolved variations in the X-ray photon absorption across a given sample are employed to generate an image. The main component of this technique, defining its spatial resolution, is given by the Fresnel zone plate (FZP) employed to focus the monochromatic X-rays generated by the synchrotron. Depending on the properties of the FZP, spatial resolutions down to sub-10 nm can be achieved (Rösner *et al.*, 2018 ▸), with typical values on the order of 20–30 nm. An image is then formed by scanning the sample with a piezoelectric stage, and detecting the intensity of the transmitted X-rays at each point of the scan. The detection process in STXM is based on single-photon counting. The transmitted X-ray photons are detected using either a phosphor screen combined with a photomultiplier tube (PMT), or an avalanche photodiode (APD). The electrical signal generated is then processed by a discriminator, generating a digitally compatible signal if the analog voltage pulse crosses a user-defined voltage threshold.

The use of an APD as a photon detector is particularly suited for time-resolved imaging, as diodes with bandwidths above the X-ray pulse repetition rate (ranging from 100 MHz to 500 MHz depending on the specific synchrotron light source) are commercially available. Using APDs with a bandwidth higher than the repetition rate allows one to resolve X-ray photons generated from neighboring X-ray bunches. This enables, in contrast to detectors with lower bandwidths, the use of the entire filling pattern to obtain the time-resolved image, instead of having to rely on the isolated camshaft bunch or on a single-bunch filling pattern. Furthermore, with this technique it is not required that the repetition rate of the signal employed to excite the dynamics is locked to the synchrotron revolution rate (determined by the circumference of the synchrotron – typically on the order of 1 µs). For the case of the PolLux beamline of the Swiss Light Source (SLS), operating with a repetition rate of about 500 MHz, the APD installed is a Hamamatsu S12023-05, with a bandwidth of 900 MHz.

The current setup employed for the acquisition of TR-STXM images at the PolLux beamline of the SLS (Raabe *et al.*, 2008 ▸), schematically shown in Fig. 1 ▸ and described in more detail in the work by Puzic *et al.* (2010 ▸) and Finizio *et al.* (2018 ▸), is based on a field-programmable gate array (FPGA) system, which consists of a 2 GSa s^−1^ ADC (where Sa means samples) combined with a discriminator for determining whether the APD has detected a photon, followed by a demultiplexer (DEMUX) that sorts the detected photons as counts in a given counter channel dependent on the bunch where the X-ray photon was generated. The APD signal is sampled with a rate of 500 MSa s^−1^, with a sampling window of 30–50 ps, centered on the moment when the X-ray pulse illuminates the APD. In order to maintain the sampling window centered with respect to the X-ray bunches, the FPGA setup needs to be synchronized with the master clock of the synchrotron light source. This synchronization is performed by an event receiver FPGA setup (Micro-Research Finland VME-EVR-230RF), which generates the relevant timing signals used to synchronize the rest of the equipment (Puzic *et al.*, 2010 ▸). By synchronizing both the DEMUX and the electronics which generate the excitation signal to the synchrotron master clock, it is possible to obtain a time-resolved image. The synchronization also guarantees that each channel of the DEMUX corresponds to a well defined sampling point in the excitation signal being investigated.

However, the setup described above and schematically depicted in Fig. 1 ▸ has two main limitations. The first is a consequence of the synchronization to the master clock, giving rise to restrictions in the accessible frequencies, whereas the second affects the temporal resolution, which is ultimately determined by the width of the X-ray bunches generated by the synchrotron.

The first limitation stems from the requirement to have both the excitation and the DEMUX synchronized to the master clock in order to keep the DAC sampling window centered with respect to the X-ray bunches, and to define the sampling points in the signal under investigation. This implies that the repetition frequency of the excitation setup is linked to the synchrotron master clock via the relation *f*_exc_ = *M* *f*_master_/*N*, *M* ≠ *N* being an integer number and *N* a prime number equal to the number of channels in the DEMUX (Puzic *et al.*, 2010 ▸). Typical values for *N* are between 7 and 23, implying that only a limited comb of frequencies can be selected with this method.

The second limitation is given by the fact that the current detection setup assumes that the X-ray photons are generated exactly at the center of their bunch. This, however, is not the case, as the width of the X-ray bunches varies depending on various parameters of the synchrotron light source (*e.g.* optics, repetition frequency, filling pattern, *etc*.). For the case of the SLS, the typical width of the X-ray pulses in the 400 mA hybrid filling pattern is about 70 ps full width at half-maximum (FWHM). This dispersion in the X-ray photon arrival time hinders the detection of high-frequency excitations, as shown in Fig. 1 ▸(*b*), where the effect of various X-ray bunch widths on the detectable contrast for different frequencies of the excitation signals is shown. Here, it is possible to observe that an FWHM of 70 ps implies that only excitation frequencies below about 10 GHz are detectable (*i.e.* detected signal amplitude above 10% of the original signal amplitude). If frequencies higher than 10 GHz need to be detected, a possible workaround to this problem is to utilize X-ray pulses with shorter widths. This is possible by operating the synchrotron light source with low-α optics, where X-ray pulse widths down to 10 ps FWHM can be achieved (Goslawski *et al.*, 2014 ▸). This reduction of the X-ray width allows, as shown in Fig. 1 ▸(*b*), the detection of frequencies above 50 GHz (for a pulse width of 10 ps FWHM). However, the scarcity of low-α optics beam times, combined with a lower photon flux with respect to the one achievable with standard optics, motivates the development of alternative detection techniques able to overcome the frequency limitations shown in Fig. 1 ▸(*b*) whilst keeping the advantages of operating the synchrotron with standard optics.

In this work, we present a TR-STXM setup based on the measurement of the arrival time of each of the X-ray photons transmitted across the sample. This setup, which does not rely on the assumption that the X-rays are generated at the center of their bunch and does not require the synchronization of the detection setup with the synchrotron master clock, would allow for both limitations presented above to be overcome. This setup, schematically depicted in Fig. 2 ▸(*a*), relies on a fast FPGA-based time-to-digital converter (QuTAG, from QuTools GmbH) that measures the time at which the input signal (*i.e.* the voltage pulse generated by the APD) crosses a defined threshold with a sub-10 ps precision. The detection of the instant when the voltage signal crosses the user-defined threshold is performed by means of a ramp interpolator. A ramp interpolator is composed of a capacitor that is charged with a constant current. When the input signal crosses the user-defined threshold, the voltage of the capacitor is sampled with an ADC, and the time at which the crossing of the threshold occurs can be calculated from the recorded value.

As long as the probability of multi-photon events within a single X-ray bunch is sufficiently low, this setup allows for the measurement of the time-of-arrival of the X-ray photons, as the time when the APD pulse arrives at the time-to-digital converter varies depending on when the photon was generated within the X-ray pulse.

The interaction of soft X-rays with the APD used to detect them does not always follow the same mechanisms. In particular, the X-ray photon can be absorbed in different layers of the junction, leading to the generation of voltage pulses with different amplitudes. Due to this dispersion in the voltage amplitudes generated by the APD, if only the moment when the rising edge of the APD pulse crosses a user-defined threshold is measured, this would lead to a considerable error in the determination of the time-of-arrival of the X-ray photon. To better depict this issue, we have measured the time-of-arrival of 500 ps electrical pulses which reproduce the typical shape of an APD pulse of different amplitudes. The result of this measurement is shown (with the amplitude normalized to the threshold voltage selected in the time-to-digital converter) in Fig. 2 ▸(*b*) (red curve for the rising edge of the pulse, and black curve for the falling edge of the pulse). There, it is possible to observe that the measured time-of-arrival is strongly affected by the amplitude of the pulse, leading to a dispersion of the arrival times of more than 100 ps. This strong dependence on the pulse amplitude can be eliminated by measuring the moment at which the rising and the falling edges of the pulse cross the defined threshold voltage, and by calculating the average between the two times, as shown by the blue curve in Fig. 2 ▸(*b*). The calculation of the average between the crossing of the threshold voltage by the rising and falling edges of the APD pulse allowed for a reduction of the dispersion of the arrival times to a 2σ uncertainty of below 10 ps, allowing us to fully exploit the performances of the time-to-digital converter. Experimentally, this measurement is performed by utilizing two channels of the time-to-digital converter, where the signal generated by the APD is fed to both channels using equal-length cables, as shown schematically in Fig. 2 ▸(*a*). One of the two channels will measure the rising edge of the pulse, while the other will measure the falling edge.

The approach described in the previous paragraph is feasible only if the voltage pulse generated by the APD is symmetrical with respect to its center and especially if the shape of the voltage pulse is not dependent on its amplitude. From oscilloscope measurements of the voltage pulses generated by the APD installed at the PolLux STXM, shown in Fig. 3 ▸, we observed that the APD pulse comprises a first symmetric pulse followed by a sinusoidal signal at a lower voltage oscillating at a frequency of about 450 MHz. This second part of the signal is caused by the ringing of the APD/RF amplifier circuit. By selecting a threshold voltage for the time-to-digital converter which is above this low-voltage ringing (*e.g.* the threshold voltage marked by the magenta dashed line in Fig. 3 ▸), it is possible to exclusively probe the first peak, containing the information about the arrival time of the X-ray photon.

As the absolute time-of-arrival of the X-ray photon is known, it is no longer necessary for the detection setup to be synchronized with the master clock of the synchrotron. However, it is necessary to know which point of the dynamical process under investigation is being sampled by the detected photon. This implies that a synchronization between the setup employed to generate the signals used to excite the dynamical processes in the sample and the time-of-arrival detector is necessary. This synchronization, marked in Fig. 2 ▸(*a*), allows us to position the recorded photon at a given instant of the excitation signal. The recorded photon arrival times can then be divided in user-defined time bins that will then be utilized to reconstruct the recorded time series. The width of the time bins can be freely selected from the user, allowing for the choice of a small time step, but with lower statistics, or of a larger time step, but with higher statistics, and anywhere in-between.

The absence of synchronization between the time-of-arrival detector (and therefore the excitation signal) and the master clock of the synchrotron light source implies that any frequency can be selected for the excitation signal (with the exception of the harmonics and sub-harmonics of the synchrotron master clock), and the excitation signal will be uniformly sampled by the X-rays.

Another very important requirement for time-resolved imaging is the determination of the time at which the excitation of the physical process is occurring. This is usually called ‘time-zero’ or *t*_0_, and its correct determination has important consequences for the interpretation of the physical processes occurring in the sample [*e.g.* for the calculation of the inertia of magnetic domain walls (Finizio *et al.*, 2019*a* ▸), skyrmions (Finizio *et al.*, 2019*b* ▸) and of spin–orbit torque induced processes (Baumgartner *et al.*, 2017 ▸)]. However, the instant at which *t*_0_ occurs is dependent on the setup employed for the specific experiment (*e.g.* waveform generators, RF components and cables) and needs to be determined experimentally. With the existing FPGA-based setup, this is typically achieved by replacing the sample with a photodiode and injecting a pulsed signal using the same electronic setup employed for the experiments, and recording a time trace. The moment at which a signal appears will correspond to *t*_0_ (Baumgartner *et al.*, 2017 ▸). For the time-of-arrival setup described here, the measurement of *t*_0_ follows the same principle.

To investigate the feasibility and performance of the setup depicted in Fig. 2 ▸(*a*) and described above, we performed a measurement of the hybrid-mode filling pattern of the SLS. In this case, the ‘excitation signal’ we wish to measure has a frequency given by the 1.042 MHz revolution rate of the bunches in the storage ring. Therefore, the 1.042 MHz bunch marker provided by the SLS was employed as the synchronization signal for the time-of-arrival detection setup. For the measurements, an integration time of 2 s was employed, and the recorded photon arrival times were sorted with 10 ps-long time bins. The measured filling pattern is shown in Fig. 4 ▸, where a single X-ray pulse is shown in the inset of the figure.

Two different hybrid-mode filling patterns were investigated. The first, shown in Fig. 4 ▸, from now on defined as a ‘short’ filling pattern, is composed of 390 filled bunches (out of a total of 480). In the gap between two consecutive bunch trains, a single filled camshaft bunch (bunch 465 – not at the center of the gap) can be found. The second, herein defined as a ‘long’ filling pattern, is instead composed of 430 filled bunches, with the camshaft bunch located at bunch 455, at the center of the gap.

From the measured photon arrival times shown in Fig. 4 ▸, various parameters of the filling pattern can be calculated. In particular, the single X-ray pulses can be fitted with a Gaussian peak function to determine the width of the X-ray pulses. The result of this calculation, for both filling patterns, is shown in Fig. 5 ▸. Here, it is possible to observe that the width of the X-ray bunches varies within the filling pattern (and between the two measured filling patterns). This change is caused by the influence of the third-harmonic passive cavities used inside the storage ring to allow the storage of a 400 mA beam.

A striking difference between the two filling patterns can, however, be observed for the camshaft bunch. Although for the short filling pattern the width of the camshaft bunch is comparable with the width of the other bunches, in the long filling pattern it is about five times larger than the width of the other bunches. This is due to the fact that the camshaft bunch for the long filling pattern is located at the center of the gap, at which the shape of the energy potential for the electrons exhibits a double-valley feature, causing an enlargement of that specific X-ray bunch.

Aside from affecting the width of the single X-ray bunches, the third-harmonic cavities also cause a displacement of the center of the X-ray bunch with respect to the positions that would be defined from the master clock of the synchrotron. This shift of the center of the X-ray bunches provides an additional uncertainty on the arrival time, which further reduces the range of frequencies accessible with the setup described in Fig. 1 ▸. From the Gaussian peak fitting of the X-ray bunches measured with the time-of-arrival setup presented here, this shift with respect to the synchrotron master clock can be determined. The results of these calculations are shown in Fig. 6 ▸.

Here it is possible to observe that the influence of the third-harmonic cavities causes a monotonically increasing delay of the center of the X-ray bunch with respect to the master clock. This offset has a similar behavior for both filling patterns and reaches up to 250 ps.

The measured filling pattern parameters were compared with streak-camera measurements of the same filling patterns, which allowed us to determine a temporal resolution on the order of 20–30 ps. This already constitutes a substantial improvement with respect to the temporal resolution of about 100 ps available with the setup shown in Fig. 1 ▸(*a*). Further improvements in the conditioning of the APD pulse are expected to bring the temporal resolution of this setup down to about 10–15 ps, providing comparable performances with low-α optics operation whilst still maintaining the high photon flux of the normal optics operation.

Up to this point, the operation of the time-of-arrival setup presented in this work was presented for a point measurement of different filling patterns of the SLS. To demonstrate that this setup can be employed for the acquisition of TR-STXM images, we performed a proof-of-principle measurement of the spin wave emission from a microstructured synthetic antiferromagnetic structure excited by an RF magnetic field. The TR-STXM image from a nanostructured antenna on a CoFeB/Py synthetic antiferromagnetic structure is provided in the supporting information; the field of view in the image is 7.5 µm × 7.5 µm and the spin waves are excited by a frequency of about 570 MHz.

In conclusion, we have presented a new concept for TR-STXM imaging based on the measurement of the time-of-arrival of the X-ray photons generated by the synchrotron light source. With this setup, operation of the excitation and detection setup does not require synchronization with the synchrotron master clock, removing any limitation on the choice of the excitation frequency. Furthermore, the temporal resolution of the setup is no longer defined by the width of the X-ray pulses generated by the synchrotron, facilitating temporal resolutions only possible up to now with low-α optics operation. With this setup, it will be possible to extend the accessible frequency range of TR-STXM imaging with standard optics operation of the synchrotron to frequencies above 10 GHz, providing an important tool for the investigation of faster dynamical processes such as those occurring in antiferromagnetic (and synthetic antiferromagnetic) materials.

## Supplementary Material

TR-STXM image of the emission of spin waves from a nanostructured antenna on a synthetic antiferromagnetic structure. DOI: 10.1107/S1600577520007262/mo5221sup1.gif

## Figures and Tables

**Figure 1:**
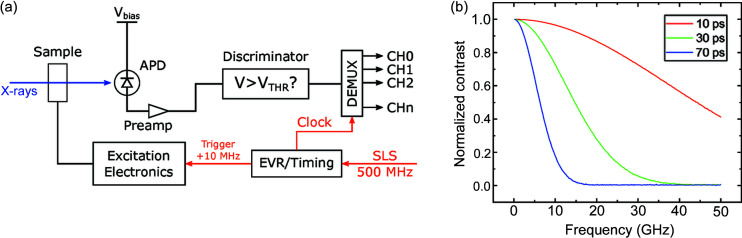
(*a*) Simplified schematic design of the time-resolved setup currently installed at PolLux. The entire setup is synchronized to the SLS 500 MHz master clock, from which all of the timing and reference signals (marked in red) are derived. (*b*) Calculated loss of contrast for a TR-STXM image as a function of the frequency of the excitation and of the full width at half-maximum (FWHM) of the X-ray pulses for the setup shown in (*a*). The contrast was normalized to the contrast visible at a frequency of 10 MHz, and it is assumed that the X-rays are generated exactly at the center of the bunches.

**Figure 2:**
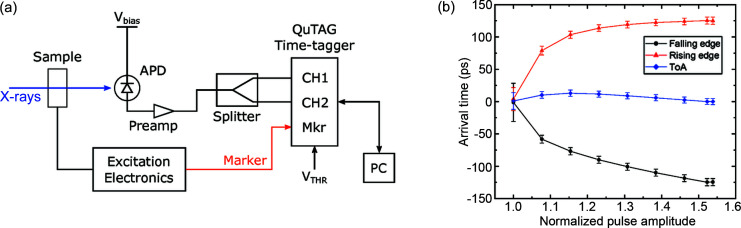
(*a*) Simplified schematic design of the time-resolved setup proposed in this work, based on the determination of the time-of-arrival of the X-ray photons. In this case, the setup is not synchronized with the synchrotron master clock, but only to the electronics employed to generate the excitation signal. (*b*) Influence of the amplitude of a reference voltage pulse (normalized to the threshold voltage *V*_THR_ selected in the time-to-digital converter) on the measured arrival time.

**Figure 3:**
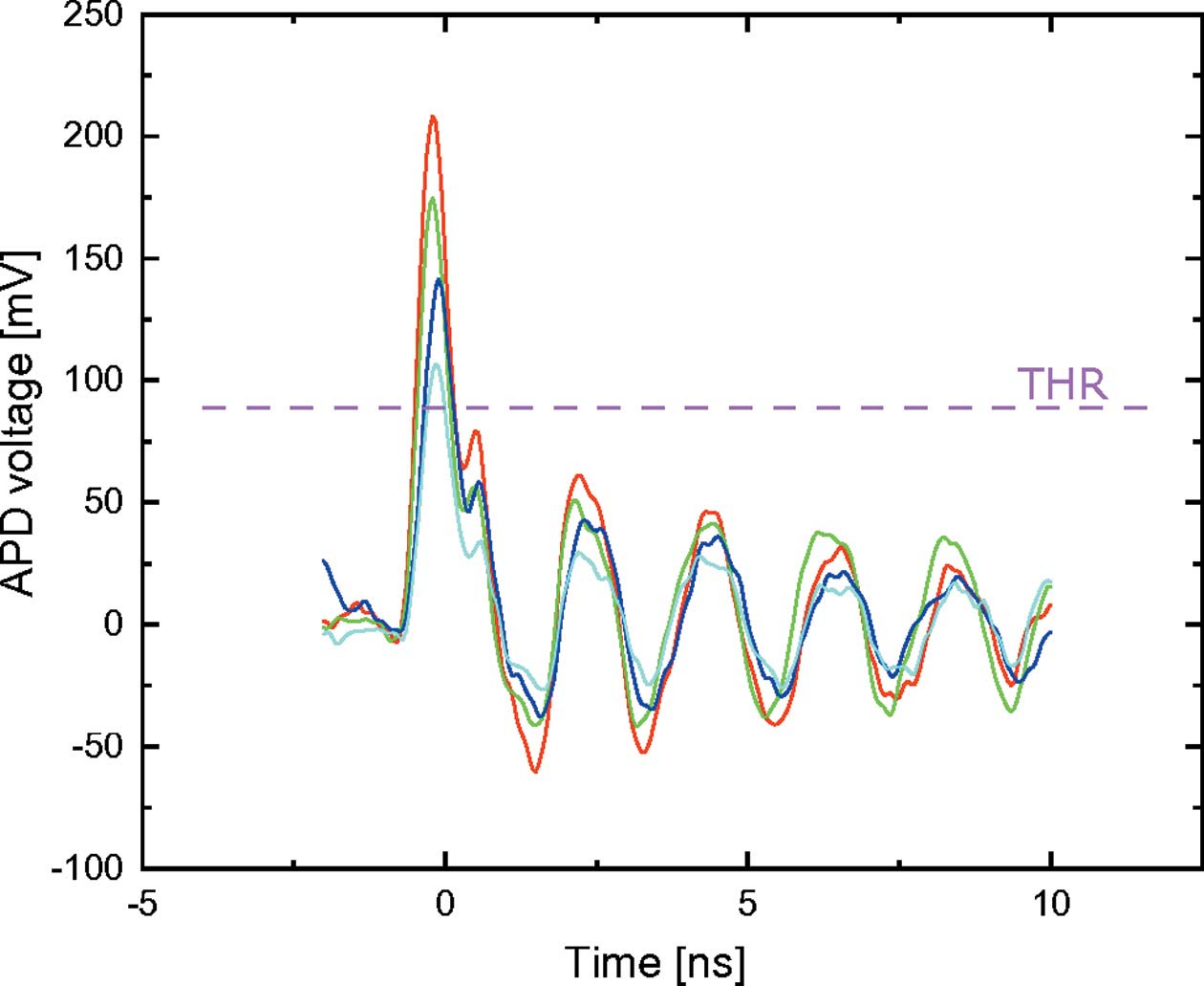
Oscilloscope trace of various APD pulses generated by the Hamamatsu S12023-05 APD installed at the PolLux endstation when exposed to a 1 keV X-ray photon beam. The maximum amplitude of the APD pulse is marked by the red curve. A significant distribution of amplitudes can be observed but, importantly, the shape of the pulse does not change with its amplitude, and comprises a first symmetric peak followed by a lower voltage sinusoidal signal caused by the ringing of the APD/RF amplifier circuit. By selecting the threshold of the time-to-digital converter to be above this sinusoidal signal (*e.g.* the threshold defined by the magenta dashed line), we can reliably determine the center of the first pulse and extract the photon arrival time from it.

**Figure 4:**
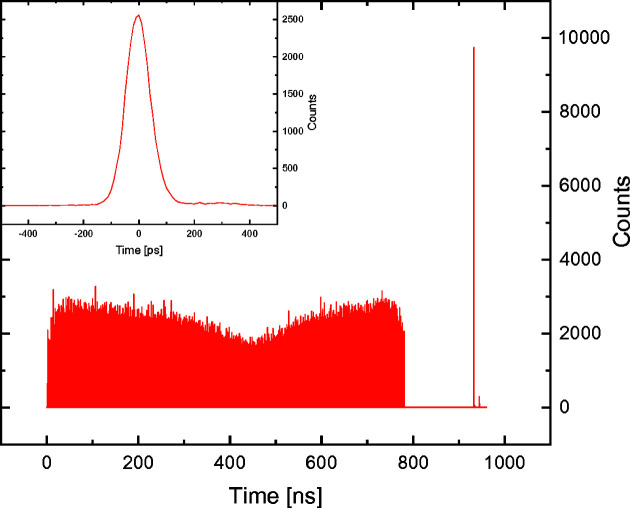
Hybrid-mode filling pattern (with camshaft) of the SLS measured with the time-of-arrival setup presented in this work. The inset shows the measurement of the 20th bunch in the filling pattern.

**Figure 5:**
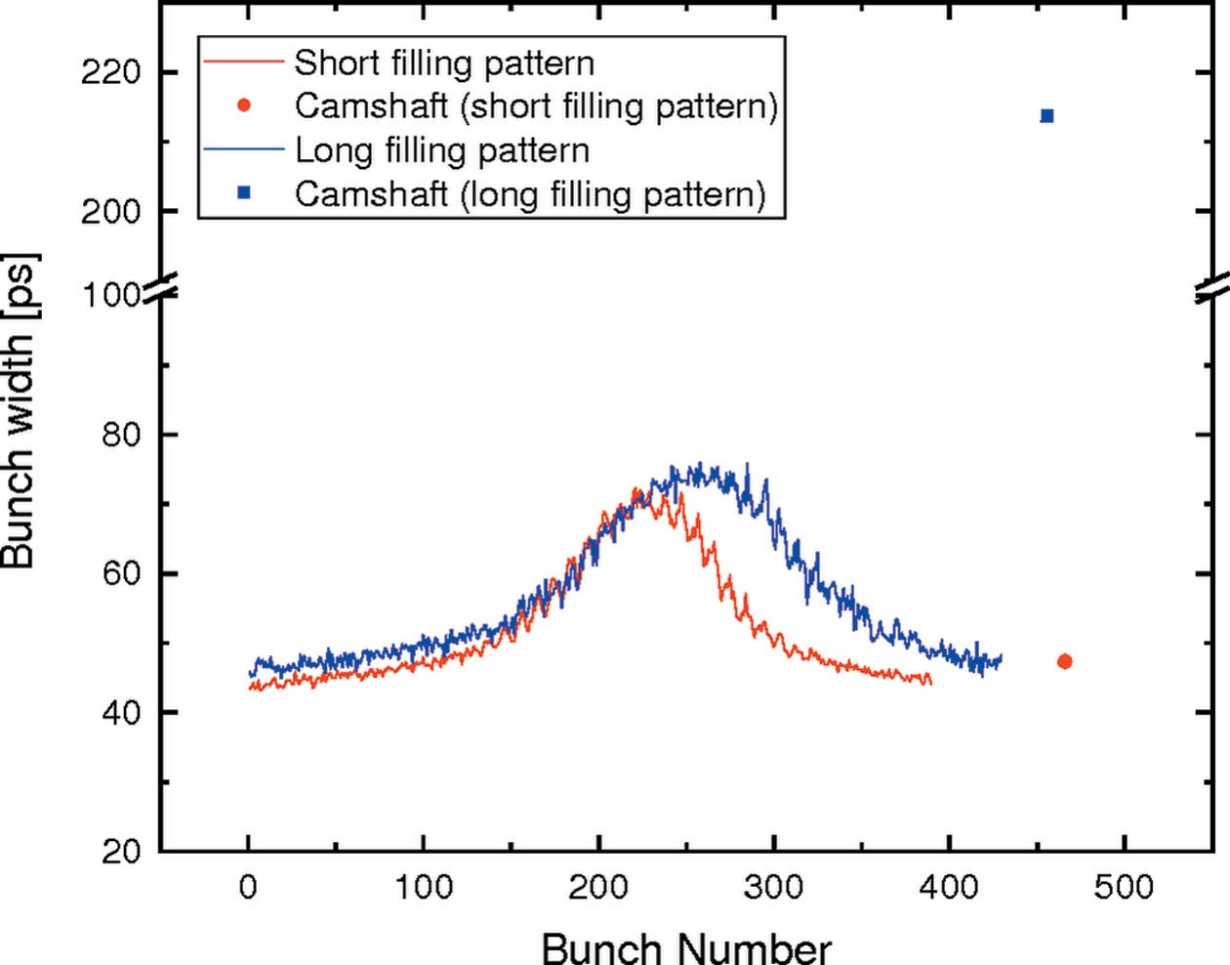
1σ width of the X-ray bunches in the filling pattern for two different filling patterns of the SLS. A variation in the width of the X-ray bunches within the multibunch section of the filling pattern, caused by the influence of the third-harmonic passive cavities inside the storage ring, can be observed. A substantial variation between the widths of the camshaft pulses of the two filling patterns can also be observed.

**Figure 6:**
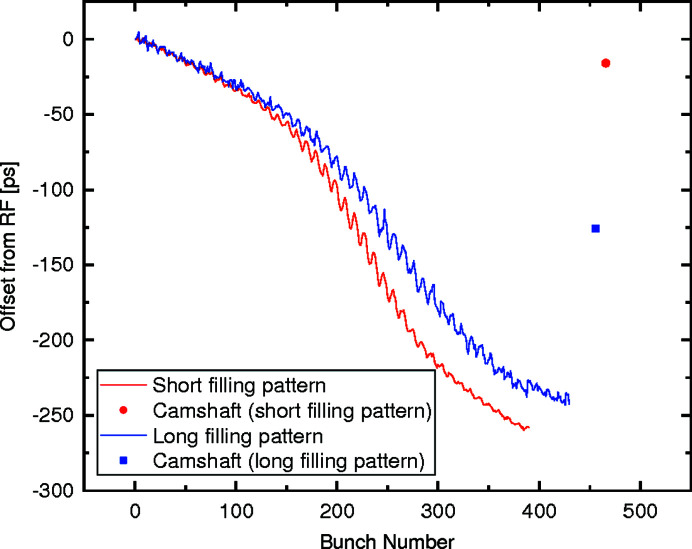
Offset of the center position of the X-ray bunches with respect to the synchrotron master clock. The change in the offset is caused by the third-harmonic passive cavities inside the storage ring.
