# Supplementary figures and images for: Time-of-arrival detection for time-resolved scanning transmission X-ray microscopy imaging
Source: J Synchrotron Radiat. 2020 Jul 14;27(Pt 5):1320–5. doi: 10.1107/S1600577520007262 (PMC7467344; doi:10.1107/S1600577520007262)

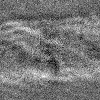

Supplement: Supplementary file 1 [file s-27-01320-sup1.gif]
